# A robust and effective framework for 3D scene reconstruction and high-quality rendering in nasal endoscopy surgery

**DOI:** 10.3389/fnbot.2025.1630728

**Published:** 2025-06-27

**Authors:** Xueqin Ji, Shuting Zhao, Di Liu, Feng Wang, Xinrong Chen

**Affiliations:** ^1^The Third School of Clinical Medicine, Ningxia Medical University, Yinchuan, China; ^2^Department of Ultrasound, Peking University First Hospital Ningxia Women and Children's Hospital, Yinchuan, China; ^3^Fudan University Academy for Engineering and Technology, Shanghai, China; ^4^Shanghai Key Laboratory of Medical Imaging Computing and Computer Assisted Intervention, Shanghai, China; ^5^Department of Hepatobiliary Surgery, General Hospital of Ningxia Medical University, Yinchuan, China

**Keywords:** nasal endoscopy, 3D reconstruction, 3D Gaussian Splatting, diffusion model, anti-aliasing

## Abstract

In nasal endoscopic surgery, the narrow nasal cavity restricts the surgical field of view and the manipulation of surgical instruments. Therefore, precise real-time intraoperative navigation, which can provide precise 3D information, plays a crucial role in avoiding critical areas with dense blood vessels and nerves. Although significant progress has been made in endoscopic 3D reconstruction methods, their application in nasal scenarios still faces numerous challenges. On the one hand, there is a lack of high-quality, annotated nasal endoscopy datasets. On the other hand, issues such as motion blur and soft tissue deformations complicate the nasal endoscopy reconstruction process. To tackle these challenges, a series of nasal endoscopy examination videos are collected, and the pose information for each frame is recorded. Additionally, a novel model named Mip-EndoGS is proposed, which integrates 3D Gaussian Splatting for reconstruction and rendering and a diffusion module to reduce image blurring in endoscopic data. Meanwhile, by incorporating an adaptive low-pass filter into the rendering pipeline, the aliasing artifacts (jagged edges) are mitigated, which occur during the rendering process. Extensive quantitative and visual experiments show that the proposed model is capable of reconstructing 3D scenes within the nasal cavity in real-time, thereby offering surgeons more detailed and precise information about the surgical scene. Moreover, the proposed approach holds great potential for integration with AR-based surgical navigation systems to enhance intraoperative guidance.

## 1 Introduction

The demand for endoscopes in transnasal surgery is growing, both for endoscopic examination and endoscopic surgery. For example, according to statistics, rhinosinusitis (RS), an inflammatory disease of the nasal cavity and paranasal sinuses, affects approximately one-six of adults in the United States, resulting in over 30 million diagnoses annually (Wyler and Mallon, 2019; Rosenfeld et al., 2015). Functional endoscopic sinus surgery (FESS), a common method for treating RS, involves inserting a slender endoscope into the nasal cavity to enter the sinus. The endoscope that enters the cavity provides the doctor with a clear field of view, which helps to accurately locate the lesion.

Endoscopy, compared to CT imaging, not only has a lower cost and no radiation, but also better real-time performance, which helps doctors accurately understand the relationship between target lesions and critical anatomical structures (Münzer et al., 2018; Pownell et al., 1997). However, mainstream monocular endoscopy cannot obtain depth information about the internal structure of the nasal cavity, which limits its application in endoscopic examination and endoscopic surgery. Therefore, surface reconstruction from endoscopic sequences enables doctors to obtain 3D information of the internal structure of the nasal cavity, which will better facilitate examination decisions and guide surgical operations.

Structure from Motion (SfM) (Snavely et al., 2006) and Simultaneous Localization and Mapping (SLAM) (Grasa et al., 2013; Mur-Artal et al., 2015) are widely used in depth estimation of endoscopic images, which recover 3D structures by tracking the position of feature points in different images. Widya et al. (2019) investigated how to utilize SfM to overcome the challenge of reconstructing gastric shapes from texture-limited endoscopic images. Leonard et al. (2016) studied an image-enhanced endoscopic navigation method based on the SfM algorithm to improve the accuracy and safety of functional endoscopic sinus surgery. Wang et al. (2020) proposed a bronchoscope enhancement scheme based on visual SLAM, which achieved the reconstruction of feature point models and improved navigation performance; (Mahmoud et al., 2017) successfully stabilized the tracking of endoscope position by combining monocular endoscopy with ORB-SLAM, and successfully repositioned it after tracking loss.

In recent years, neural rendering (Kato et al., 2018; Tewari et al., 2020; Mildenhall et al., 2021) used differentiable rendering and neural networks, surpassing the limited performance of traditional 3D reconstruction. For instance, Wang et al. (2022) utilized dynamic neural radiance fields to represent deformable surgical scenes and explored the potential of neural rendering in 3D reconstruction of surgical scenes. Batlle et al. (2023) introduced LightNeus, which combines neural implicit surface reconstruction technology with photometric models of light sources to achieve 3D reconstruction of the entire colon segment. Chen P. et al. (2024) first utilized Neural Radiance Fields (NeRF) (Mildenhall et al., 2021) to achieve 3D reconstruction of dynamic cystoscopic examination scenes, which can recover scenes under limited perspectives and features, alleviating texture loss problem that traditional algorithms may encounter.

Furthermore, doctors can observe lesion areas from different perspectives through 3D reconstruction using videos obtained from endoscopic examinations, aiding in formulating more precise surgical plans and predicting surgical difficulty and risks. During surgery, the real-time rendering of the 3D scene inside the nasal cavity can be achieved through the posture of the endoscopic camera, providing additional perspective and depth information, which enables doctors to perform cutting, suturing, and other operations more accurately. However, the application of traditional methods in 3D reconstruction of nasal endoscopy has certain limitations. For example, geometry-based reconstruction techniques, such as SfM (Snavely et al., 2006; Widya et al., 2019; Leonard et al., 2016; Schonberger and Frahm, 2016) and SLAM (Grasa et al., 2013; Wang et al., 2020; Mahmoud et al., 2017; Mur-Artal et al., 2015), often struggle to accurately capture feature points in complex nasal scenes with rich vascular networks and lack of distinct textures, resulting in sparse reconstruction. Additionally, endoscopic images may be affected by lighting effects and lens jitter, leading to image blurring and making reconstruction more complex. The emerging technology based on NeRF (Wang et al., 2022; Batlle et al., 2023; Chen P. et al., 2024) is to use implicit neural representation for volume parameterization of 3D space, which not only is the flexibility poor, but also has slow inference speed, greatly reducing the real-time performance of intraoperative surgery.

Therefore, in this paper, a nasal endoscope reconstruction model, Mip-EndoGS is proposed. Specifically, building upon the foundation of the 3D Gaussian Splatting model (3D-GS) (Kerbl et al., 2023), we employ a diffusion model to alleviate the impact of dynamic blurring in endoscopic images on the reconstruction results. In addition, an adaptive low-pass filter is introduced to reduce aliasing artifacts during the rendering process. We collect a dataset of high-definition surgical videos of nasal examinations performed by professional physicians, recording the spatial position of each frame. Subsequently, we apply the proposed Mip-EndoGS model to this dataset, achieving high-quality and real-time rendering of 3D nasal endoscopic scenes. The main contributions of this paper are as follows.

1. A nasal endoscopy reconstruction model, Mip-EndoGS, is proposed to achieve high-quality 3D reconstruction of nasal endoscopy, which integrates a diffusion module into the 3D Gaussian Splatting framework to remove blur from endoscopic images.
2. An adaptive low-pass filter is embedded into the Gaussian rendering pipeline to overcome aliasing artifacts, which achieves realistic 3D reconstruction of nasal endoscopy scenes.
3. Extensive quantitative and qualitative experiments are conducted to validate the proposed model's effectiveness in reconstructing and rendering nasal endoscopy scenes.

## 2 Materials and methods

### 2.1 Nasal endoscopy dataset

The nasal endoscopy dataset, NasED, is constructed by our own. There are 16 subjects with a total of 51 video segments. The data is collected using XION 4K endoscope and NDI optical surgical navigation system. The videos record the process from the inferior and middle nasal meatus to the pharyngeal orifice of the eustachian tube, capturing multi-angle shots of the internal nasal structures, such as the middle and inferior turbinates. Furthermore, the video data is preprocessed into nasal endoscopy examination images with a resolution of 1280 x 720, totaling over 30,000 frames.

### 2.2 Method architecture

**Figure 1:**
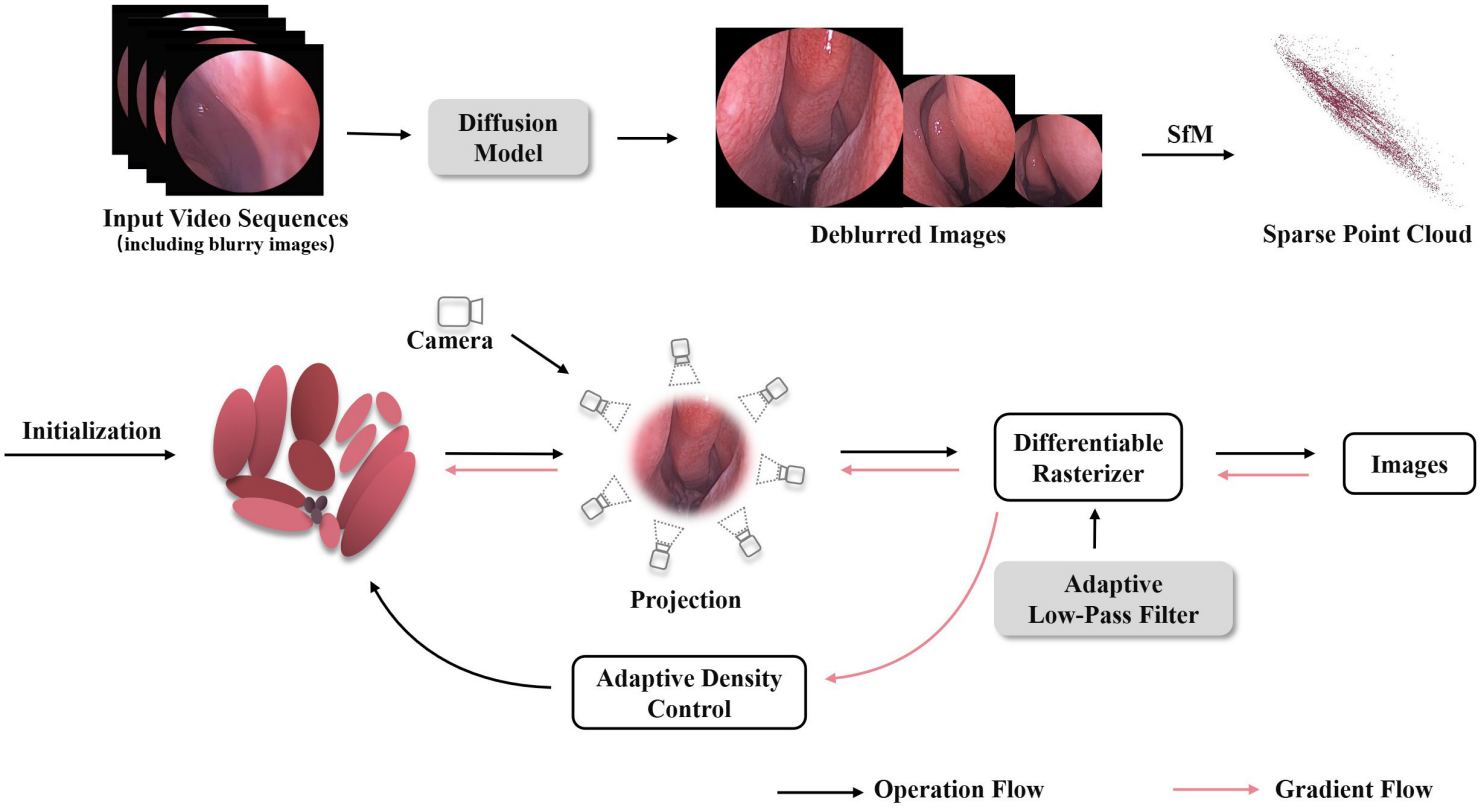
The overview of our Mip-EndoGS pipeline. Firstly, relatively blurry views are processed by the diffusion module for deblurring processing. Subsequently, the improved image sequence is processed through SfM to obtain sparse 3D point clouds and camera poses. Then, The generated point clouds and camera poses are fed into the Gaussian splatting pipeline for fast differentiable rendering. Adaptive low-pass filtering is applied during splatting to reduce aliasing and improve the quality of 3D nasal endoscopy reconstruction.

The proposed high fidelity 3D reconstruction and rendering model framework is shown in Figure 1, which comprises two stages, image enhancement based on the diffusion model and 3D-GS differentiable rendering using an adaptive low-pass filter. In the first stage, we uniformly sample several endoscopic views from the endoscopic video in chronological order and select relatively blurry views as input to the diffusion module (Chen Z. et al., 2024) for deblurring processing. Subsequently, the deblurred views are merged with the original ones to obtain an image-enhanced sequence of endoscopic images. In the second stage, the optimized image sequence is processed through Structure-from-Motion (SfM) (Snavely et al., 2006) algorithms to obtain sparse 3D point clouds and camera poses. These generated point clouds and camera poses are then inputted into the Gaussian splatting pipeline for fast differentiable rasterization rendering (Kerbl et al., 2023). In the splatting rendering process, adaptive low-pass filtering is designed to overcome aliasing issues, thereby achieving high-quality 3D reconstruction of nasal endoscopic scenes.

#### 2.2.1 Image enhancement based on diffusion models

In the process of reconstructing 3D nasal cavity based on endoscopic video, the factors may potentially affect the quality of the images, such as the blurriness caused by the mutual compression of nasal tissues or induced by the dynamic movement of the endoscope. Meanwhile, the potential noise can affect feature extraction between consecutive frames. Therefore, the advanced HI-Diff (Chen Z. et al., 2024) method is employed to denoise the captured nasal endoscopic images, which combines the Transformer based reconstruction module with the traditional diffusion model, and utilizes hierarchical concentration modules (Zamir et al., 2022) to enhance the deblurring process.

**Figure 2:**
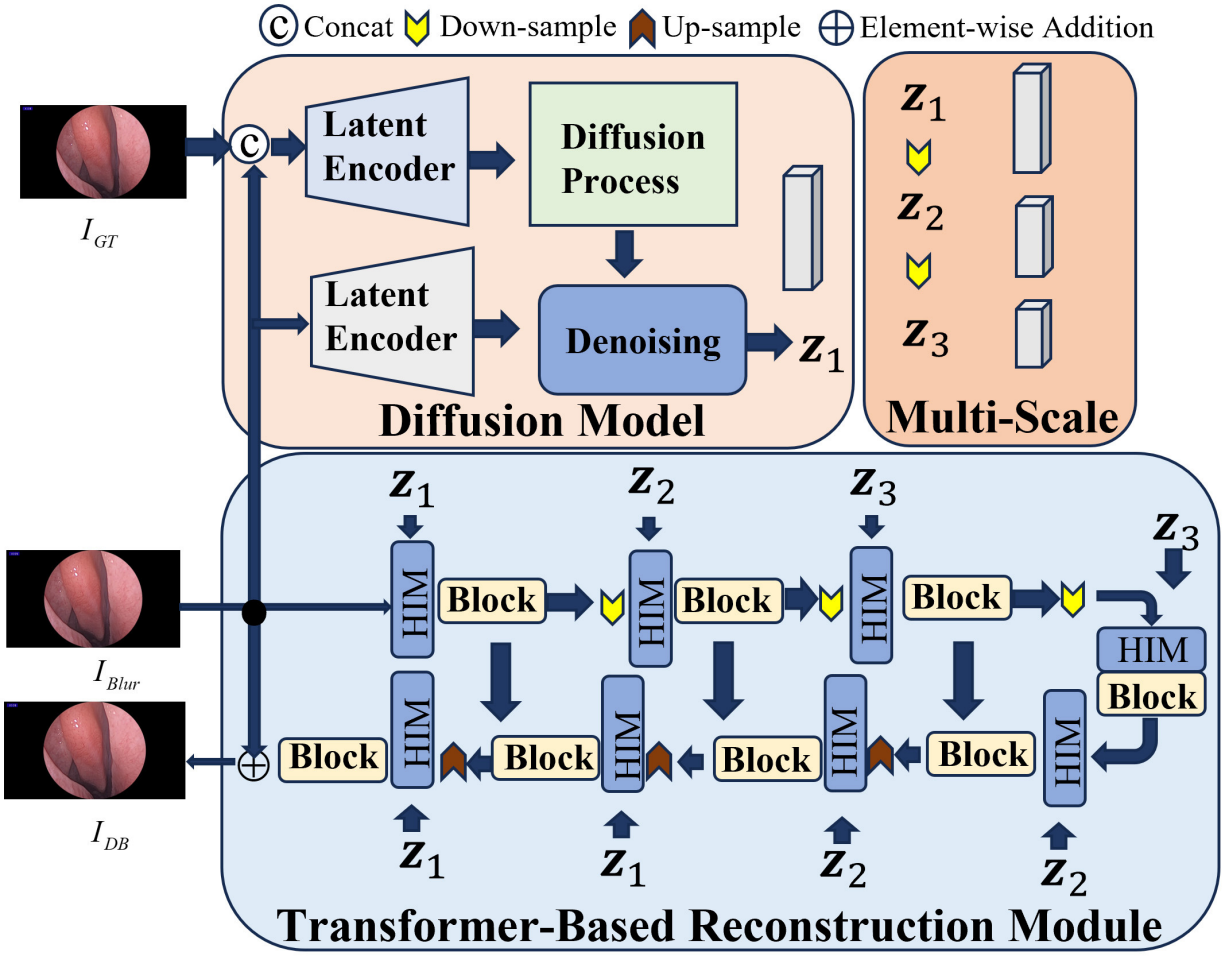
The framework of HI-Diff. Blurry views are fed into HI-Diff, which performs deblurring to recover clearer and more detailed structures.

The overall framework of HI-Diff deblurring is illustrated in Figure 2. During the training process, given the input blurry image *I*_Blur_ and its corresponding ground truth image *I*_GT_, there are two identical latent encoders (LE) (Rombach et al., 2022) employed to process both images. Specifically, the concatenated form of the blurry image *I*_Blur_ and its corresponding ground truth image *I*_GT_ is first fed into one of the latent encoders to extract the prior features *v*. Simultaneously, the blurry image *I*_Blur_ is fed into another LE to be mapped to a conditional latent vector *p*. The specific procedure is as follows:


(1) $$\begin{array}{l} v=f_{LE1}\left(I_{GT}\text{©}I_{Blur}\right)\text{,} \end{array}$$

(2) $$\begin{array}{l} p=f_{LE2}\left(I_{Blur}\right)\text{,} \end{array}$$

Where *f*_*LE*1_ and *f*_*LE*2_ denote the mappings of the images into high-dimensional space, and © represents the concatenation of the two images.

Subsequently, adhering to the procedures outlined in the diffusion model, the prior features are subjected to the addition of random Gaussian noise before being inputted into the denoising network, resulting in *v*_*T*_. Concurrently, the conditional latent vector *p* is also fed into the denoising network. This denoising network, conditioned on both inputs, proceeds to predict the ultimate prior features *v*_1_. The detailed process unfolds as follows:


(3) $$\begin{array}{l} v_{T}=f_{diffusion}\left(v\right)\text{,} \end{array}$$

(4) $$\begin{array}{l} v_{1}=f_{denoising}\left(v_{T},p\right)\text{,} \end{array}$$

Where *f*_*diffusion*_ denotes the process of adding noise to the prior features *v*, and *f*_*denoising*_ represents the neural network. This network takes the vector *v*_*T*_, which has been augmented with random noise, along with *p* as inputs, to predict the prior features *v*_1_.

Moreover, due to the non-uniform blurriness induced by the dynamic motion of the endoscope, relying solely on a single scale of prior features may not adequately accommodate complex blurring scenarios. Hence, to acquire multi-scale prior features capable of adapting to various scales of intermediate features, *v*_1_ is downsampled twice. The specific procedure unfolds as follows:


(5) $$\begin{array}{l} v_{2}=f_{down-sample}\left(v_{1}\right)\text{,} \end{array}$$

(6) $$\begin{array}{l} v_{3}=f_{down-sample}\left(v_{2}\right)\text{.} \end{array}$$

For the Transformer-based reconstruction module, given the input blurry image *I*_*Blur*_, the reconstruction module undergoes multiple rounds of upsampling and downsampling before reconstructing the clear image *I*_*DB*_. Furthermore, at each feature extraction stage, a hierarchical concentration module is positioned ahead of both the encoder and decoder, which serves to fuse the intermediate features *X*_*in*_ from the Transformer with the multi-scale prior features *v*_1_,*v*_2_,*v*_3_ from the diffusion model through cross-attention fusion. Its purpose is to enhance the deblurring process of the Transformer.

During the testing phase, we replace the ground truth image *I*_*GT*_ with randomly generated Gaussian noise. The blurry image *I*_*Blur*_ is then fed into the diffusion model to obtain the prior features *v*_1_. Subsequently, these prior features are utilized to enhance the blurry image within the Transformer-based reconstruction module, resulting in the generation of high-quality, clear images.

#### 2.2.2 Differentiable rendering through 3D Gaussians Splatting

To achieve fast differentiable rasterization rendering through 3D-GS splatting (Kerbl et al., 2023), the sparse point clouds along with their corresponding camera poses are required, which can be estimated by tracking feature points across multiple images based on Structure-from-Motion (SfM)(Snavely et al., 2006). Based on these point clouds, a set of Gaussian functions using the position mean μ and covariance matrix *A* is defined. To enhance the representation of the scene, each Gaussian function is equipped with opacity σ and a set of spherical harmonic functions. By introducing this anisotropic 3D Gaussian distribution as a high-quality and unstructured representation of the radiation field, not only can the model compactly represent 3D scenes, but flexible optimization processes are also supported. Specifically, the probability density function of the Gaussian model is as follows (Zwicker et al., 2001b):


(7) $$\begin{array}{l} N\left(\text{x}\right)=e^{-\frac{1}{2}{\left(\text{x}-\mu \right)}^{T}A^{-1}\left(\text{x}-\mu \right)}\text{,} \end{array}$$

where *A* can be decomposed into two more specific components, the quaternion *r* and the 3D-vector *s*. Then, these components are transformed into the corresponding rotation and scaling matrices *R* and *S*. Therefore, the covariance matrix *A* can be represented as:


(8) $$\begin{array}{l} A=RSS^{T}R^{T}\text{.} \end{array}$$

During the rendering stage, Gaussian elements need to be projected into the rendering space (Zwicker et al., 2001a). Through view transformation, the new covariance matrix in the camera coordinate system can be calculated as follows:


(9) $$\begin{array}{l} A^{\prime }=JWAW^{T}J^{T}\text{,} \end{array}$$

where *J* is the Jacobian matrix approximating the affine transformation of the projection.

Additionally, these Gaussian elements are projected onto the imaging plane according to the observation matrix, and colors are blended based on opacity and depth (Kopanas et al., 2022, 2021). Therefore, the final color *C*(*p*) of the *p*-th pixel can be represented by blending M ordered points overlapping the pixel:


(10) $$\begin{array}{l} C\left(p\right)=\underset{i\in M}{\sum }T_{i}\alpha_{i}c_{i}\text{,} \end{array}$$

with


$$\begin{array}{l} \alpha_{i}=\sigma_{i}e^{-\frac{1}{2}{\left(p-\mu_{i}\right)}^{T}A^{\prime }\left(p-\mu_{i}\right)}\text{and}T_{i}=\overset{i-1}{\underset{j=1}{\prod }}\left(1-\alpha_{j}\right)\text{.} \end{array}$$

*T*_*i*_ is the transmittance, *c*_*i*_ represents the color of the Gaussian element along the direction of the ray, and μ_*i*_ denotes the projected 2D *UV* coordinates of the 3D Gaussians.

Efficient rendering and depth sorting are achieved through a fast tile-based differentiable raster izer. Additionally, the α-blending technique is introduced to adjust opacity σ and scale parameter *S* through a sigmoid function, which ensures that the image synthesis maintains higher visual quality.

The rendered scene is compared with the corresponding image to calculate the loss for rapid backpropagation. The loss function consists of $L_{1}$ loss and Structural Similarity Index Measure (SSIM), balanced by adjusting the weighting factor λ. It is expressed as follows:


(11) $$\begin{array}{l} L=\left(1-\lambda \right)L_{1}+\lambda L_{\text{D-SSIM}}\text{.} \end{array}$$

Here, the Stochastic Gradient Descent (SGD) algorithm is utilized to optimize the model parameters iteratively to minimize the loss function (Fridovich-Keil et al., 2022). To further optimize the model, adaptive density control is implemented to adjust the number and density of Gaussian elements for better scene representation. The introduction of transparency threshold ϵ_α_ and position gradient threshold τ_*pos*_ is used to control the addition and removal of Gaussian elements. The introduction of this adaptive control allows the method to better adapt to the geometric complexity of nasal endoscopy scenes.

#### 2.2.3 Adaptive low-pass filter

In the rendering process, aliasing is a fundamental issue, as rendered images are usually sampled based on discrete raster grids, inevitably leading to visual artifacts such as jagged edges along object contours and Moir é fringes in textures. A similar phenomenon occurs when splashing elliptical Gaussian, when the scene is reconstructed and rendered at a lower sampling rate.

Previous research (Hu et al., 2023; Yu et al., 2023; Barron et al., 2021) attempts to mitigate aliasing effects generally by prefiltering (Heckbert, 1989; Mueller et al., 1998) and super-sampling techniques (Cook, 1986). For example, the EWA volume reconstruction (Zwicker et al., 2001a) introduces the notion of resampling filters, combining the reconstruction algorithm with a low-pass kernel. Inspired by this method, we employ an anti-aliasing filter to alleviate aliasing artifacts during nasal endoscope rendering. Building upon Equation 10, we further elaborate the rasterization formula:


(12) $$\begin{array}{l} C\left(p\right)=\overset{M}{\underset{i=1}{\sum }}c_{i}\sigma_{i}N_{i}^{\prime }\left(p\right)\overset{i-1}{\underset{j=1}{\prod }}\left(1-\sigma_{j}N_{j}^{\prime }\left(p\right)\right)\text{,} \end{array}$$

Where $N_{i}^{\prime }\left(p\right)$ represents the projection of the Gaussian distribution onto a two-dimensional plane, closely related to the two-dimensional covariance (Kopanas et al., 2021). And the 2 × 2 variance matrix *A*^′′^ can be easily obtained from the 3 × 3 matrix *A*′ by skipping the third row and column:


(13) $$\begin{array}{l} A^{\prime }=\left(\begin{array}{ccc} a & b & c\\ b & d & e\\ c & e & f \end{array}\right)\Leftrightarrow \left(\begin{array}{cc} a & b\\ b & d \end{array}\right)=A^{\prime \prime }\text{.} \end{array}$$

Following that, to simulate the diffusion effect occurring during the propagation of light rays, the scale of the 2D covariance is adjusted (Kerbl et al., 2023), for which a positive definite adjustment term is added to the original covariance matrix *A*^′′^.

The adjustment term is a scalar multiplied by the unit matrix related to the hyperparameter, by which the scale of the covariance matrix is increased and the diffusion effect of light rays is simulated. Furthermore, in the actual imaging process, the light captured by each pixel accumulates within its surface area, meaning the final image is obtained by integrating the photon energy falling on each pixel (Shirley, 2018). To achieve the actual imaging process more efficiently, the “Adaptive Low-Pass Filter” is proposed as shown in Equation 14, which adapts to different sampling rates and changes in perspective when processing endoscopic images, while maintaining the visual quality of the image.


(14) $$\begin{array}{l} N^{2D}{\left(\text{x}\right)}_{\text{low-pass}}=\sqrt{\frac{\left|A^{\prime \prime }\right|}{\left|A^{\prime \prime }+s\text{I}\right|}}e^{-\frac{1}{2}{\left(\text{x}-\mu \right)}^{T}{\left(A^{\prime \prime }+s\text{I}\right)}^{-1}\left(\text{x}-\mu \right)}\text{.} \end{array}$$

The scale parameter in the adaptive low-pass filter controls the extent of Gaussian smoothing. Intuitively, it simulates the physical diffusion of light across pixel areas due to limited resolution and sampling rates. A larger scale parameter induces stronger anti-aliasing but risks oversmoothing details, while a smaller scale parameter preserves sharpness but may cause jagged edges.

## 3 Results

In this section, the proposed method, Mip-EndoGS, is evaluated based on our nasal endoscopy dataset, NasED. Firstly, the implementation setting of Mip-EndoGS is presented. Then, we provide a detailed introduction of the metrics used in the experiment. Finally, the experimental results are showed, including both quantitative analysis and qualitative analysis.

### 3.1 Experiments setting

The NasED dataset comprises several monocular nasal endoscopy video sequences, denoted as ${\left\{H_{i}\right\}}_{i=1}^{T}$. Here, *T* represents the number of sequences, and *H*_*i*_ denotes the *i*-th sequence. Each nasal sequence is divided into several frames, denoted as ${\left\{A_{j}\right\}}_{j=1}^{M}$, where *M* is the total number of frames in the sequence, and *j* represents the index of the *j*-th frame. Hence, the *i*-th sequence and *j*-th frame's endoscopic view is represented as (*H*_*i*_, *A*_*j*_). From this dataset, we extracted four groups of video sequences: *H*_1_, *H*_2_, *H*_3_, and *H*_4_. Each group consists of randomly sampled consecutive 100-frame views, totaling 400 frames. Each sequence is split into 90% training data and 10% testing data. These video sequences are captured by a monocular camera, covering the internal structures of the nasal cavity and sinuses.

In the diffusion module, we adhere to experimental settings consistent with HI-Diff and load weights trained on the GoPro (Nah et al., 2017) synthetic dataset for image denoising. Sparse point clouds and camera poses are obtained through COLMAP (Snavely et al., 2006; Schonberger and Frahm, 2016). The parameters of the Gaussian rendering pipeline (Kerbl et al., 2023) follow the original method settings, except for the changes in the number of iterations. The scale parameter in the adaptive low-pass filter is set to 0.3, and the learning rate is set to 1e-4. The network is trained on an NVIDIA RTX A6000 device.

### 3.2 Metric

To conduct a thorough assessment of our experimental results, various methods are employed to evaluate the reconstruction outcomes, primarily comprising quantitative analysis and qualitative assessment through visualization. For quantitative analysis, we utilized several commonly used evaluation metrics, including the Structural Similarity Index Measure (SSIM), Peak Signal-to-Noise Ratio (PSNR), and Learned Perceptual Image Patch Similarity (LPIPS).

The computation of SSIM is as follows, which measures the similarity between two images in terms of brightness, contrast, and structure:


(15) $$\begin{array}{l} \mathrm{SSIM}\left(x,y\right)=\frac{\left(2\mu_{x}\mu_{y}+c_{1}\right)\left(2\sigma_{xy}+c_{2}\right)}{\left(\mu_{x}^{2}+\mu_{y}^{2}+c_{1}\right)\left(\sigma_{x}^{2}+\sigma_{y}^{2}+c_{2}\right)}\text{,} \end{array}$$

where *x* and *y* represent the two images to be compared, μ_*x*_ and μ_*y*_ denote their mean intensities, $\sigma_{x}^{2}$ and $\sigma_{y}^{2}$ represent their variances, σ_*xy*_ indicates their covariance, and *c*_1_ and *c*_2_ are variables used to stabilize the denominator.

The definition of PSNR is as follows:


(16) $$\begin{array}{l} \mathrm{PSNR}=20\cdot \log_{10}\left(\frac{\text{MA}{\text{X}}_{I}}{\sqrt{\text{MSE}}}\right)\text{,} \end{array}$$

where MAX_*I*_ represents the maximum possible pixel value of the image, and MSE is the mean squared error between the reconstructed image and the reference image.

LPIPS employs deep learning models to evaluate the perceptual similarity between images, capturing texture and structural differences crucial for human visual perception:


(17) $$\begin{array}{l} \text{LPIPS}\left(x,y\right)=\sum_{l}w_{l}\cdot ||\phi_{l}\left(x\right)-\phi_{l}\left(y\right)|{|}_{2}\text{,} \end{array}$$

where ϕ_*l*_(*x*) and ϕ_*l*_(*y*) represent the feature maps of images *x* and *y* extracted by a pre-trained deep neural network at layer *l*, and *w*_*l*_ is a learned weight used to emphasize the importance of each layer's contribution to perceptual similarity.

By applying these metrics, we can quantitatively analyze the quality of our image reconstructions.

### 3.3 Results analysis

#### 3.3.1 Evaluation on full resolution

**Figure 3:**
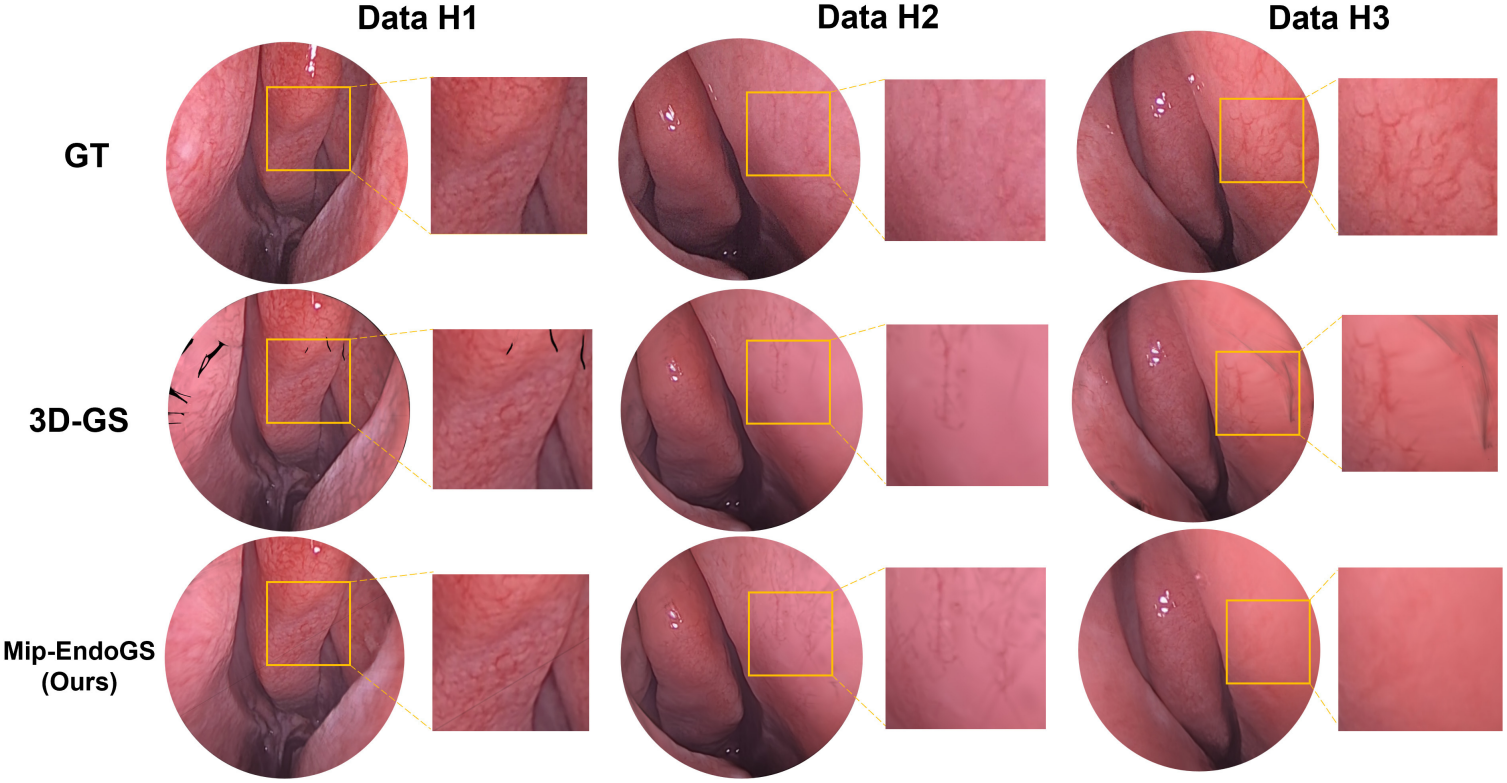
Qualitative results presentation on different video sequences.

To validate the model's strong generalization capability, we selected sequences from different subjects. The results are presented in Table 1 and Figure 3. Figure 3 illustrates the rendering effects of sequences *H*_1_, *H*_2_ and *H*_3_ after 40k iterations of model training.

**Table 1:** Quantitative comparison of rendering quality on different video sequences.

**Method**	**H1**			**H2**			**H3**		
	**PSNR**	**SSIM**	**LPIPS**	**PSNR**	**SSIM**	**LPIPS**	**PSNR**	**SSIM**	**LPIPS**
3D-GS	26.52	**0.942**	**0.114**	32.67	0.953	0.137	26.34	0.925	0.156
Mip-EndoGS	**27.50**	0.936	**0.114**	**35.95**	**0.971**	**0.022**	**30.16**	**0.934**	**0.145**

The best results are in bold.

A comparison with the Ground Truth reveals that despite the narrow field of view and lack of texture in nasal endoscopic views, our method renders nasal structures distinctly with clear textures. Compared to the original 3D-GS method, the proposed approach demonstrates higher stability, effectively reducing issues such as significant aliasing, artifacts, and distortions in certain areas observed in the output of 3D-GS. Quantitative evaluation through Table 1 shows notable improvements in the PSNR metrics across all four datasets. Additionally, except for *H*_1_, the SSIM and LPIPS metrics for the other three datasets also achieve superior results.

#### 3.3.2 Compared with COLMAP

**Figure 4:**
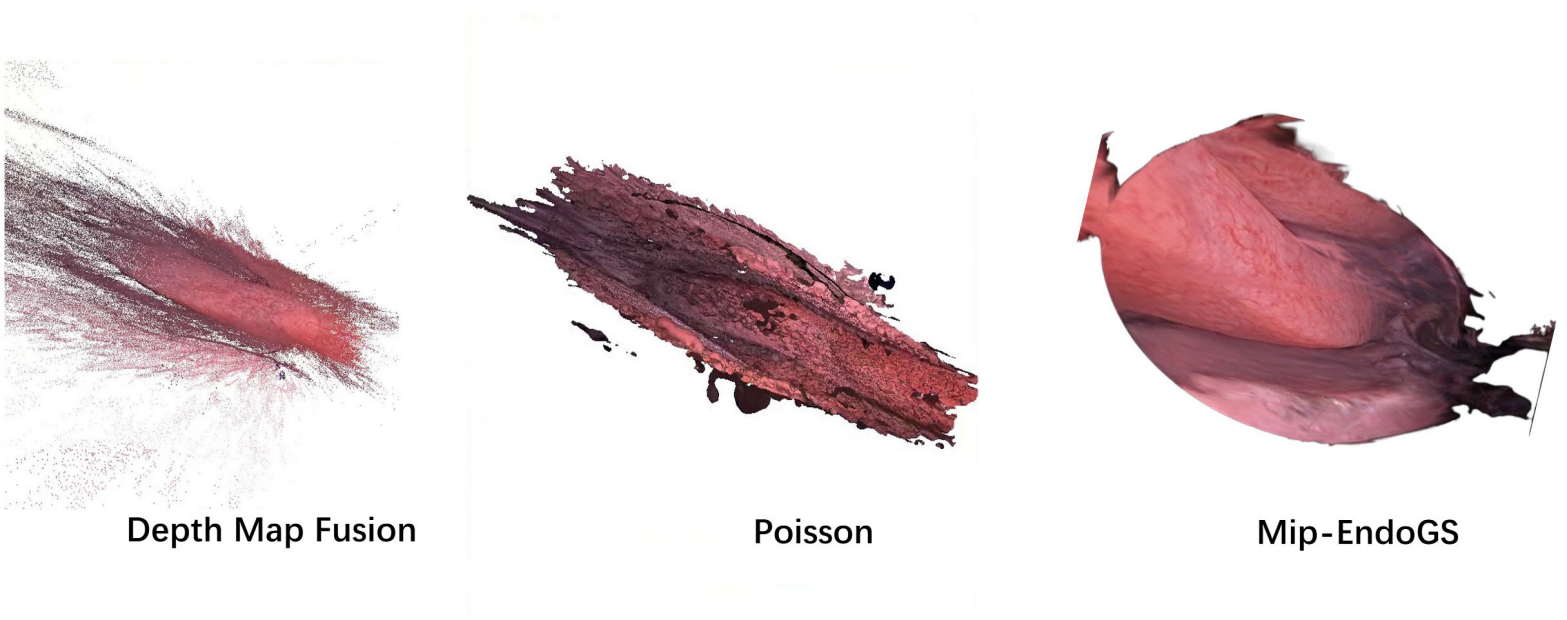
Comparison of surface reconstruction from ours and COLMAP.

The proposed method, Mip-EndoGS, is compared with the current mainstream reconstruction methods, Depth Map Fusion (Merrell et al., 2007) and the Poisson method (Kazhdan and Hoppe, 2013) in COLMAP. and the visual results are shown in Figure 4.

Data *H*_1_ is utilized in this evaluation. Evidently, the nasal endoscopic scenes reconstruct with Mip-EndoGS exhibit more realistic and smoother features, demonstrating excellent visual outcomes. Apart from comparison with COLMAP, we also attempt reconstruction using methods based on neural radiance fields such as NeRF (Mildenhall et al., 2021) and Neuraludf (Long et al., 2023). However, due to the unique characteristics of nasal structures, these methods all fail.

#### 3.3.3 Evaluation on different iterations

**Figure 5:**
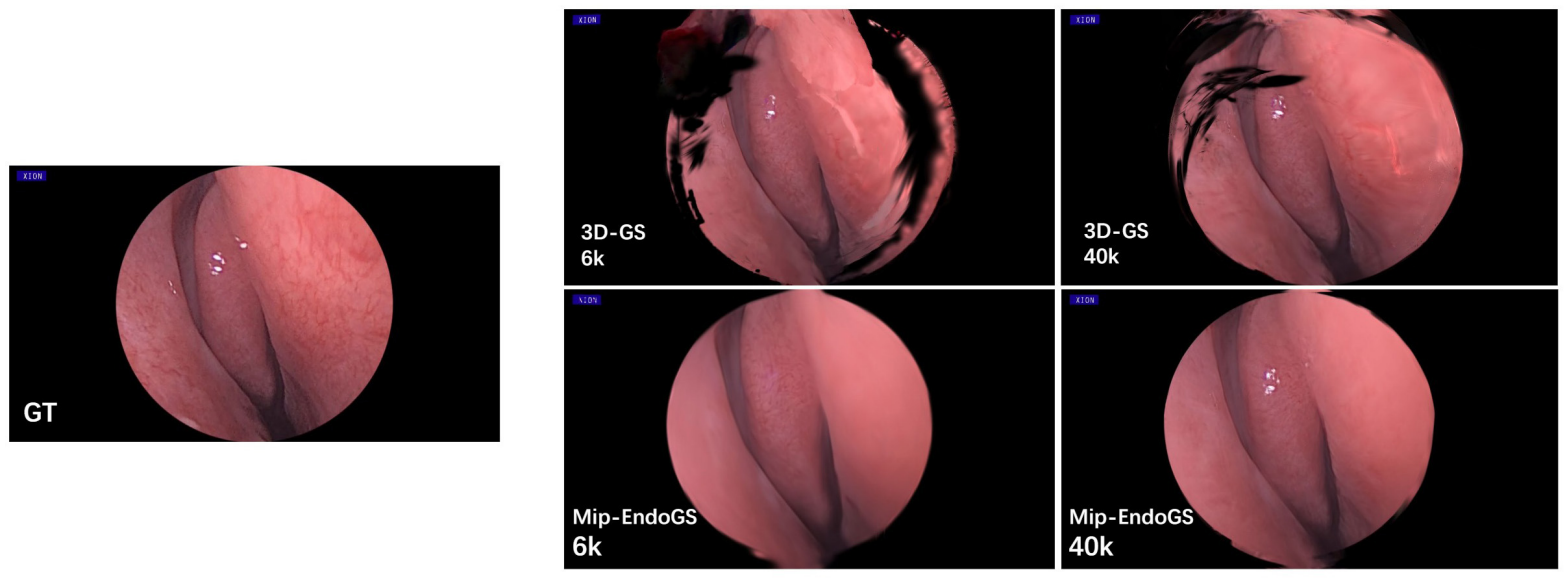
Qualitative comparison of different outcomes after 6k and 40k iterations.

Table 2 and Figure 5 respectively present the quantitative results and visual effects of 3D-GS and Mip-EndoGS at 6k and 40k iterations (evaluated using *H*_3_ data), which shows that our model is capable of capturing the structures within the nasal cavity clearly after 6k iterations, with the PSNR metric significantly outperforming the rendering results of 3D-GS at the same iteration count.

**Table 2:** Quantitative comparison of different outcomes after 6k and 40k iterations.

**Method**	**6k**			**40k**		
	**PSNR**	**Train time**	**FPS**	**PSNR**	**Train time**	**FPS**
3D-GS	20.63	1 m 53 s	112	26.34	12 m 23 s	91
Mip-EndoGS	**27.49**	3 m 14 s	105	**30.16**	14m	86

The best results are in bold.

In terms of time, the proposed model only takes around 3 minutes for 6k iterations, less than 1/4 of the time required for 40k iterations. This shorter training time, coupled with clear structural representation, is crucial for real-time surgical navigation. Moreover, although the addition of the diffusion module slightly affects the training time and rendering speed of our model, it still achieves real-time rendering capability.

#### 3.3.4 Evaluation on various resolution

**Figure 6:**
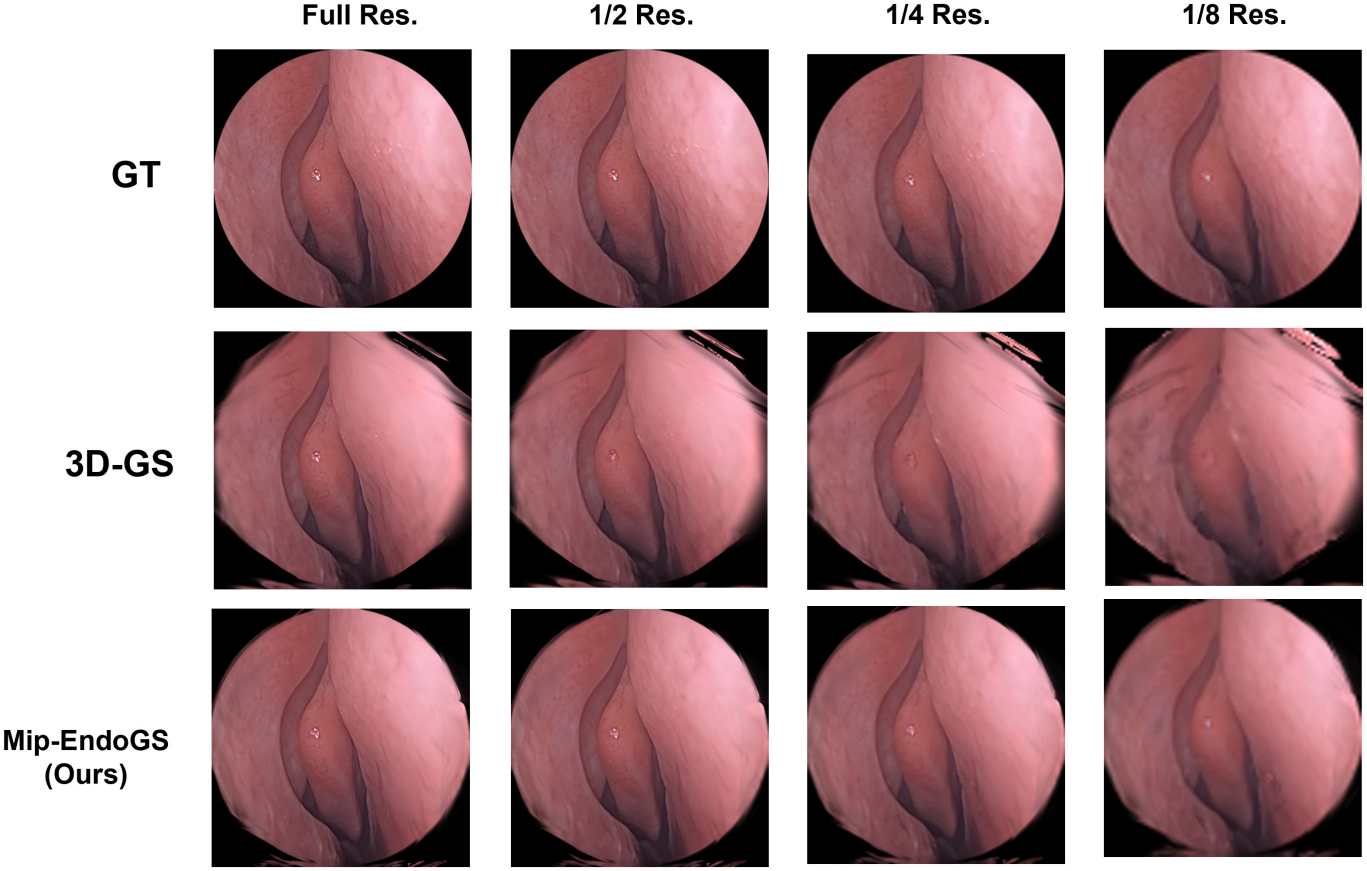
Qualitative comparison of single-scale training and multi-scale testing.

To simulate the reconstruction effects of scenes at low sampling rates, the original data are downsampled to obtain datasets with resolutions reduced to 1/2, 1/4, and 1/8 of the original resolution. We train the model on the original resolution data and render on the downsampled datasets accordingly. The quantitative evaluation is conducted using *H*_1_ and *H*_4_ data (as shown in Table 3), where the proposed method outperforms 3D-GS in rendering quality at lower resolutions. The visual results for *H*_4_ are shown in Figure 6, where the proposed method produces the higher fidelity imagery without apparent artifacts and aliasing.

**Table 3:** Quantitative comparison of single-scale training and multi-scale testing.

**Dataset**	**Method**	**PSNR**					**SSIM**					**LPIPS**				
		**Full Res**.	**1/2 Res**.	**1/4 Res**.	**1/8 Res**.	**Avg**.	**Full Res**.	**1/2 Res**.	**1/4 Res**.	**1/8 Res**.	**Avg**.	**Full Res**.	**1/2 Res**.	**1/4 Res**.	**1/8 Res**.	**Avg**.
H1	3D-GS	26.52	26.50	26.82	29.44	27.32	**0.942**	**0.936**	0.910	**0.962**	0.938	**0.114**	0.085	0.089	0.052	0.085
	Mip-EndoGS	**27.50**	**27.79**	**28.66**	**31.10**	**28.74**	0.936	0.927	**0.933**	0.954	**0.938**	**0.114**	**0.084**	**0.066**	**0.046**	**0.076**
H4	3D-GS	20.81	20.69	19.87	20.14	20.38	**0.883**	**0.854**	0.835	0.812	0.846	0.197	0.203	0.202	0.230	0.208
	Mip-EndoGS	**21.33**	**21.24**	**21.14**	**23.73**	**21.86**	0.872	0.832	**0.837**	**0.876**	**0.854**	**0.191**	**0.191**	**0.180**	**0.125**	**0.172**

The best results are in bold.

## 4 Discussion

Nasal endoscopic scene reconstruction contributes to a comprehensive understanding of the surgical environment, precise surgical localization, and critical information provision for minimally invasive procedures. However, nasal cavity structures are not only narrow and intricate, but also lack distinctive texture features. Additionally, the influence of endoscopic lighting often makes it challenging to capture nasal cavity structural characteristics. Moreover, the quality of views collected by endoscopy is difficult to guarantee, often resulting in blurriness and contamination.

To address these issues, this paper introduces an advanced nasal endoscopic reconstruction model, Mip-EndoGS, which enables real-time rendering of scenes and synthesis of new viewpoints during surgery by pre-training before surgery. The proposed method consists of two parts, an image enhancement module based on diffusion models and a 3D-GS differentiable rendering pipeline using adaptive low-pass filters. The image enhancement module used in this paper integrates a Transformer-based reconstruction module with traditional diffusion models and employs a hierarchical attention mechanism to enhance the deblurring process of the Transformer, achieving denoising effects on collected nasal endoscopic images. For the differentiable rendering pipeline based on 3D-GS, we embed an adaptive low-pass filter to overcome aliasing artifacts, which simulates the diffusion effect during light propagation and integrates the photon energy falling on each pixel to adapt to changes in sampling rates and viewpoints.

The proposed method can reconstruct highly realistic nasal endoscopic scenes on the NasED dataset. As shown in the experimental results, the reconstructed nasal structures are distinct with clear textures. Compared to the original 3D-GS, the proposed method demonstrates higher stability, effectively alleviating issues such as aliasing artifacts and distortions during rendering. The high-quality reconstruction results can provide more accurate 3D information, assisting surgeons in diagnosis and reducing surgical risks.

In practice, this task will be combined with motion tracking technology to create a more convenient and intelligent surgical navigation workspace. Additionally, with the development of augmented reality and virtual display technologies, doctors can perform detailed surgical simulations preoperatively and provide real-time three-dimensional views intraoperatively. Such capabilities are particularly valuable in complex or minimally invasive procedures, where accurate spatial perception is critical. These technological advancements can provide doctors with more intuitive and easier-to-use surgical assistance and offer patients higher-quality medical services.

However, certain limitations still exist, such as the occlusions caused by medical instruments and the hands of the surgeon during surgery, as well as deformations of nasal tissues from various angles. These failure cases highlight the need for further optimization in complex surgical environments. To address these challenges, more intelligent surgical planning and navigation technologies are urgently needed.

## 5 Conclusion

In this work, a novel method, Mip-EndoGS, is proposed to reconstruct the scene of nasal endoscopy. The method combines the diffusion model and 3D Gaussian model, initially employing the diffusion model for deblurring and then achieving high-quality real-time rendering using 3D Gaussian. Additionally, we collect high-definition surgical video datasets from nasal examinations performed by professional doctors and validate the proposed method on this dataset. In the experiment, the proposed method demonstrates superior performance in both quantitative assessment and visual analysis. In the future, we plan not only to expand this dataset but also to further refine the related algorithms.

## Data Availability

The raw data supporting the conclusions of this article will be made available by the authors upon request.
